# Three studies, three steps forward in cardiovascular care

**DOI:** 10.1007/s12471-024-01923-2

**Published:** 2024-12-11

**Authors:** Pim van der Harst

**Affiliations:** Department of Cardiology, Division of Heart and Lungs, University Medical Centre Utrecht, Utrecht, The Netherlands

This edition of the Netherlands Heart Journal highlights three studies that each take an important step forward in improving cardiovascular care.

The first study, by Gingele and colleagues, introduces the Maastricht Decompensation Questionnaire (MDQ), a simple tool designed to identify fluid overload in heart failure patients using common signs and symptoms [1]. Intended for use in outpatient clinics and potentially integrable into eHealth tools, the MDQ is a promising development in early detection and management of heart failure decompensation. While further validation in larger trials is needed, the initial findings support its practical use in clinical settings.

The second study, by Hart and colleagues, presents the first Dutch experience with the FlowTriever system for catheter-directed thrombectomy in intermediate-high and high-risk pulmonary embolism [2]. This technique, used by interventional cardiologists at UMC Utrecht, provides a minimally invasive alternative for patients in whom systemic thrombolysis is contraindicated or ineffective. With excellent technical success rates and manageable complication risks, the study demonstrates the feasibility of FlowTriever in the Netherlands and provides a basis for expanding its use.

Finally, the study by Wester and colleagues. investigates sex differences in outcomes after coronary artery bypass grafting (CABG) using data of 51,753 Dutch patients in the Netherlands Heart Registration (NHR) [3]. Women undergoing CABG presented with more complex risk profiles and received fewer arterial grafts than men. Although sex itself was not a predictor of mid-term mortality in the overall cohort, younger women (< 70 years) experienced worse outcomes compared to men. These findings highlight the need to better understand and address sex-specific differences in preoperative risk profiles and outcomes in cardiovascular surgery.

All three studies offer valuable insights and encourage to improve on personalised patient care. From improving tools for managing heart failure to exploring new treatments for pulmonary embolism and creating awareness of sex differences in surgical outcomes, these three studies help us move closer to better outcomes for our patients.
